# Penehyclidine hydrochloride inhibits renal ischemia/reperfusion-induced acute lung injury by activating the Nrf2 pathway

**DOI:** 10.18632/aging.103444

**Published:** 2020-07-11

**Authors:** Zhaohui Liu, Yan Li, Lili Yu, Yulin Chang, Jingui Yu

**Affiliations:** 1 Department of Anesthesiology, Qilu Hospital, Cheeloo College of Medicine, Shandong University, Jinan, Shandong, China; 2 Department of Anesthesiology, Cangzhou Central Hospital, Cangzhou, Hebei, China

**Keywords:** penehyclidine hydrochloride, renal ischemia/reperfusion, oxidative stress, acute lung injury, inflammation

## Abstract

The nuclear factor (NF)-κB and NOD-like receptor protein 3 (NLRP3) pathways promote inflammatory signaling that injures the kidneys, whereas the nuclear factor erythroid 2-related factor 2 (Nrf2) pathway promotes anti-inflammatory signaling that inhibits oxidative damage. Penehyclidine hydrochloride (PHC) inhibits NF-κB and activates Nrf2 signaling. We investigated whether PHC induces communication between the Nrf2 and NF-κB/NLRP3 pathways, thereby protecting against renal ischemia/reperfusion (rI/R)-induced lung inflammation. Rat alveolar macrophages (NR8383 cells) were stimulated for 24 h with PHC with or without brusatol (a Nrf2 antagonist), after which they were treated for 4 h with tert-butyl hydroperoxide (10 mM). PHC Nrf2-dependently alleviated tert-butyl hydroperoxide-induced reactive oxygen species production in alveolar macrophages. Additionally, wild-type and Nrf2^−/−^ rats were each divided into four groups: (1) sham, (2) PHC (1 mg/kg), (3) rI/R and (4) rI/R + PHC (1 mg/kg). PHC markedly induced the Nrf2 and adenosine monophosphate-activated protein kinase pathways and suppressed rI/R-induced NF-κB and NLRP3 activation in the lungs. Nrf2 deficiency diminished the ability of PHC to ameliorate rI/R-induced histopathological alterations and reactive oxygen species release in the lungs; however, PHC inhibited NLRP3 signaling Nrf2-dependently, while it inhibited NF-κB signaling Nrf2-independently. Our findings demonstrate the beneficial effects of PHC on rI/R-induced lung inflammation.

## INTRODUCTION

Redox equilibrium is critical for the existence of aerobionts [1]. Redox equilibrium is preserved when the production of free radicals and oxidants (particularly reactive oxygen species [ROS]) is balanced by their elimination through internal antioxidants [2]. However, detrimental stimuli may induce excessive ROS release, thus overpowering these antioxidants and damaging proteins, lipids and DNA [3]. Oxidative stress is associated with the development of diabetes mellitus, chronic kidney disease, neurodegenerative disease, chronic lung disease and other diseases [4–7]. Inflammatory responses, which are well-recognized contributors to different disorders, are closely associated with oxidative stress [8]. Thus, exploring antioxidant cascades that can be activated by internal or external substances may foster the development of therapies for various human diseases.

Nuclear factor erythroid 2-related factor 2 (Nrf2) is a crucial antioxidative transcription factor [9]. In the absence of stimulation, Kelch-like ECH-associated protein 1 (Keap1) retains Nrf2 in the cytoplasm in an inactive state [10]. However, oxidative stress can induce the detachment of Nrf2 from Keap1 via multiple protein kinase signals [11, 12]. The intracellular energy transducer adenosine monophosphate-activated protein kinase (AMPK) participates in both energy metabolism and redox equilibrium [13], and may activate Nrf2 through signaling cascades involving glycogen synthase kinase 3β (GSK3β) and Akt [14, 15]. Once released from Keap1, Nrf2 shifts to the nucleus and binds to antioxidant response elements (AREs) in various cellular defensive genes, including glutamate-cysteine ligase modifier subunit (GCLM), glutamate-cysteine ligase catalytic subunit (GCLC), heme oxygenase-1 (HO-1) and NADPH quinone oxidoreductase 1 (NQO1) [16]. The initiation of antioxidant pathways protects animals from oxidative damage and inflammatory responses simultaneously [17, 18].

The interactions between inflammatory responses and oxidative stress have been confirmed in various studies [19, 20]. Pro-inflammatory signals such as nuclear factor kappa B (NF-κB) and the NOD-like receptor protein 3 (NLRP3) inflammasome promote both inflammatory responses and oxidative stress [21, 22]. The NLRP3 inflammasome can be induced by various “hazard excitants”, including ROS and ATP [23, 24]. NLRP3 induces pro-inflammatory proteins such as interleukin (IL)-18 and IL-1β by activating caspase-1 and the adaptor protein ASC [25]. Interestingly, NF-κB not only functions as a typical pro-inflammatory signal, but also participates in the early stage of NLRP3 activation [26]. Moreover, there is an interdependent relationship between the pro-inflammatory effects of NF-κB/NLRP3 and the antioxidative effects of Nrf2 [27].

**Figure 1:**
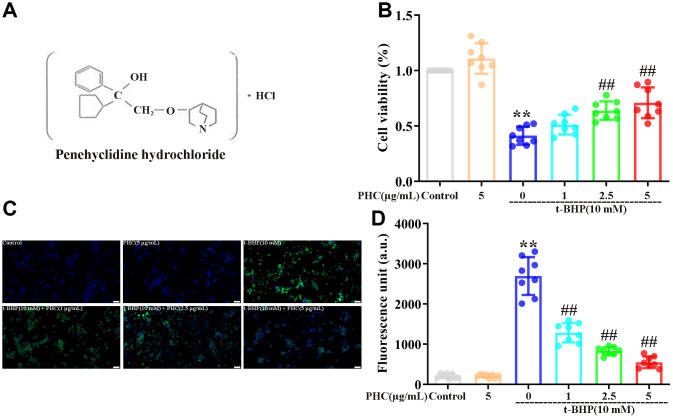
**Effects of PHC on t-BHP-induced oxidative damage in NR8383 cells.** (**A**) The structure of PHC. (**B**) NR8383 cells were stimulated with PHC (1, 2.5, 5 μg/mL) for 24 h, and then exposed to t-BHP (10 mM) for 4 h. A CCK8 analysis was used to measure cell viability. (**C**, **D**) NR8383 cells were stimulated with PHC (1, 2.5, 5 μg/mL) for 24 h, stained with DCFH-DA (5 μM) for 40 min and treated with t-BHP (10 mM) for 5 min to trigger ROS production. A fluorescence microplate reader and microscope (original magnification 200×) were used to measure ROS levels. Data are presented as the mean ± standard deviation (S.D.) (n = 8, Scale bar: 50 μm). *P < 0.05, **P < 0.01 vs. the control group. ^#^*P* < 0.05, ^##^*P* < 0.01 vs. the t-BHP group.

Renal ischemia/reperfusion (rI/R)-induced lung inflammation, a major disease with limited remedies and high lethality, is strongly dependent on oxidative stress and inflammatory responses [28–31]. We previously explored the detrimental effects of NF-κB, mitogen-activated protein kinase, toll-like receptor 4 and NLRP3 and the protective functions of HO-1 in rI/R-induced lung inflammation [29–31]. Penehyclidine hydrochloride (PHC, Figure 1A), an intravenous anesthetic with sedative and analgesic properties, is an effective NF-κB inhibitor and Nrf2 activator that can suppress caspase-3 activation and apoptosis [32–35]. Nevertheless, it is not known how Nrf2 communicates with NF-κB/NLRP3 and whether PHC defends against rI/R-induced lung inflammation by promoting this crosstalk. Considering that non-infectious stimuli such as rI/R-induced inflammatory protein secretion drastically increase inflammatory responses in the lungs, in the current experiment, we explored the ability of PHC to prevent rI/R serum-induced alveolar macrophage injury and rI/R-induced lung inflammation in rats.

## RESULTS

### PHC attenuated t-BHP-induced ROS production and cell death in NR8383 cells in a Nrf2-dependent manner

The cytoprotective effects of PHC have been established in rat alveolar macrophages (NR8383 cells) treated with tert-butyl hydroperoxide (t-BHP) [34]. In a Cell Counting Kit 8 (CCK8) analysis, we found that t-BHP stimulated cell death in NR8383 cells, whereas pre-conditioning (24 h) with PHC inhibited this effect (Figure 1B). Administration of t-BHP also notably increased ROS production in NR8383 cells, while PHC pre-conditioning alleviated it (Figure 1C, 1D). Thus, PHC may have inhibited cytotoxicity by eliminating ROS in NR8383 cells. However, preconditioning with brusatol (a specific Nrf2 inhibitor) reversed the effects of PHC on ROS production and cell viability (Supplementary Figure 1A, 1B in Supplementary Material), indicating that the antioxidative and cytoprotective functions of PHC in t-BHP-treated NR8383 cells depended on Nrf2.

### PHC enhanced the Nrf2/AMPK pathways and relevant antioxidant enzyme production in NR8383 cells

**Figure 2:**
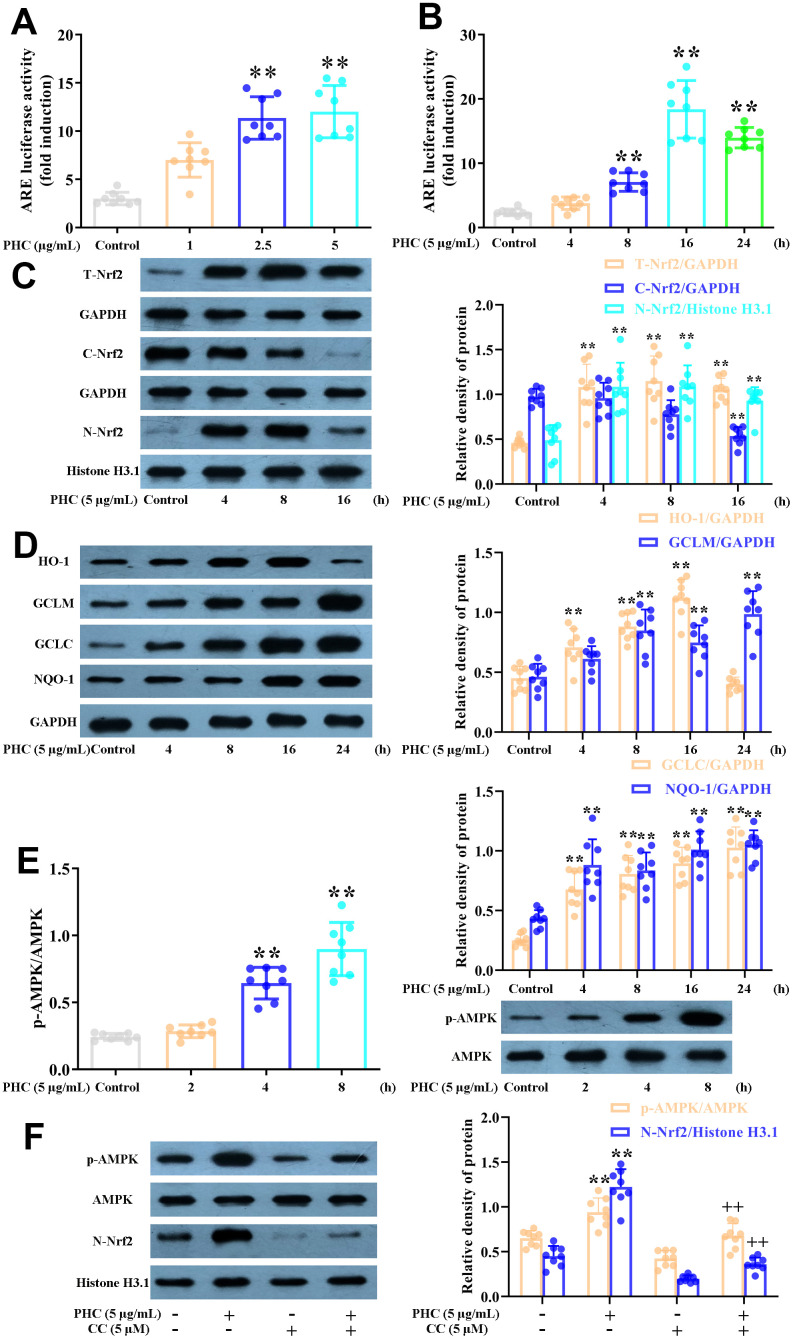
**Effects of PHC on Nrf2/AMPK signaling in NR8383 cells.** (**A**, **B**) A dual-luciferase reporter analysis system was used to measure the effects of PHC on ARE luciferase activity at different time points (4, 8, 16 or 24 h) or PHC concentrations (1, 2.5 or 5 μg/mL) in NR8383 cells. (**C**) NR8383 cells were stimulated with PHC (5 μg/mL) for different durations (4, 8 or 16 h). Western blotting was used to determine the total, nuclear and cytoplasmic concentrations of Nrf2. (**D**) NR8383 cells were stimulated with PHC (5 μg/mL) for different durations (4, 8, 16 or 24 h). Western blotting was used to measure the protein levels of NQO1, HO-1, GCLM and GCLC. (**E**) NR8383 cells were incubated with PHC (5 μg/mL) for different durations (2, 4 or 8 h). Western blotting was used to measure the protein levels of p-AMPK and AMPK. (**F**) NR8383 cells were stimulated with CC (an antagonist of AMPK, 5 μM) for 24 h, and then were exposed to PHC for 3 h. Western blotting was used to measure the nuclear concentration of Nrf2 and the protein levels of p-AMPK and AMPK. GAPDH was used as an internal control for total and cytoplasmic proteins, while histone H3.1 was used as an internal control for nuclear proteins. Data are presented as the mean ± S.D. (n = 8). *P < 0.05, **P < 0.01 vs. the control group. ^+^*P* < 0.05, ^++^*P* < 0.01 vs. the PHC alone group.

During oxidative stress, the production of antioxidant enzymes (including GCLM, HO-1, NQO1 and GCLC) induced by Nrf2 increases. As anticipated, PHC notably enhanced the luciferase reporter activity of a promoter containing an ARE (Figure 2A, 2B). Furthermore, PHC time-dependently enhanced GCLM, HO-1, NQO1, GCLC and total Nrf2 protein levels in NR8383 cells, and reduced cytoplasmic Nrf2 levels while increasing nuclear Nrf2 levels (Figure 2C, 2D).

Previous research has suggested that AMPK is an upstream inducer of the Nrf2 pathway. We discovered that PHC activated AMPK somewhat before it induced Nrf2 in NR8383 cells (Figure 2E). Moreover, the PHC-induced activation of Nrf2 was reduced when the cells were preconditioned with compound C (CC, an AMPK inhibitor) (Figure 2F). Thus, AMPK functioned as an upstream enhancer of the PHC-stimulated activation of Nrf2 in NR8383 cells.

### PHC defended NR8383 cells from rI/R serum-induced inflammatory responses through both Nrf2-dependent and -independent mechanisms

**Figure 3:**
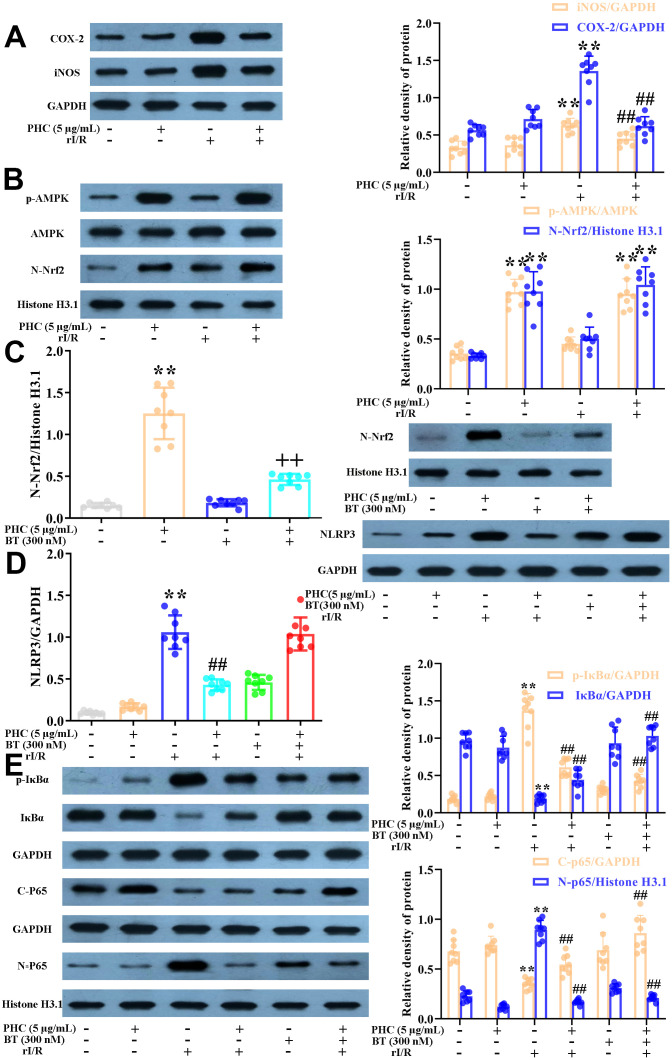
**Effects of PHC on rI/R serum-induced inflammatory reactions in NR8383 cells.** (**A**, **B**) NR8383 cells were stimulated with PHC (5 μg/mL) for 1 h and then treated with serum from rI/R rats for 24 h. Western blotting was used to measure the protein levels of COX-2 and iNOS (**A**), as well as the nuclear concentration of Nrf2 and the protein levels of AMPK and p-AMPK (**B**). (**C**) NR8383 cells were stimulated with brusatol (an antagonist of Nrf2, 300 nM) for 1 h and then exposed to PHC (5 μg/mL) for 1 h before being treated with serum from rI/R rats for 24 h. Western blotting was used to measure Nrf2 levels. (**D**) NR8383 cells were stimulated with brusatol (300 nM) for 1 h, and then exposed to PHC (5 μg/mL) for 1 h before being treated with serum from rI/R rats for 24 h. Western blotting was used to measure the protein levels of NLRP3. (**E**) NR8383 cells were stimulated with brusatol (300 nM) for 1 h and then exposed to PHC (5 μg/mL) for 1 h before being treated with serum from rI/R rats for 24 h. Western blotting was used to measure the cytoplasmic and nuclear concentrations of p65, as well as the protein levels of IκBα and p-IκBα. GAPDH was used as an internal control for total and cytoplasmic proteins, while histone H3.1 was used as an internal control for nuclear proteins. Data are presented as the mean ± S.D. (n = 8). *P < 0.05, **P < 0.01 vs. the control group. ^#^*P* < 0.05, ^##^*P* < 0.01 vs. the rI/R serum group. ^+^*P* < 0.05, ^++^*P* < 0.01 vs. the PHC alone group.

**Figure 4:**
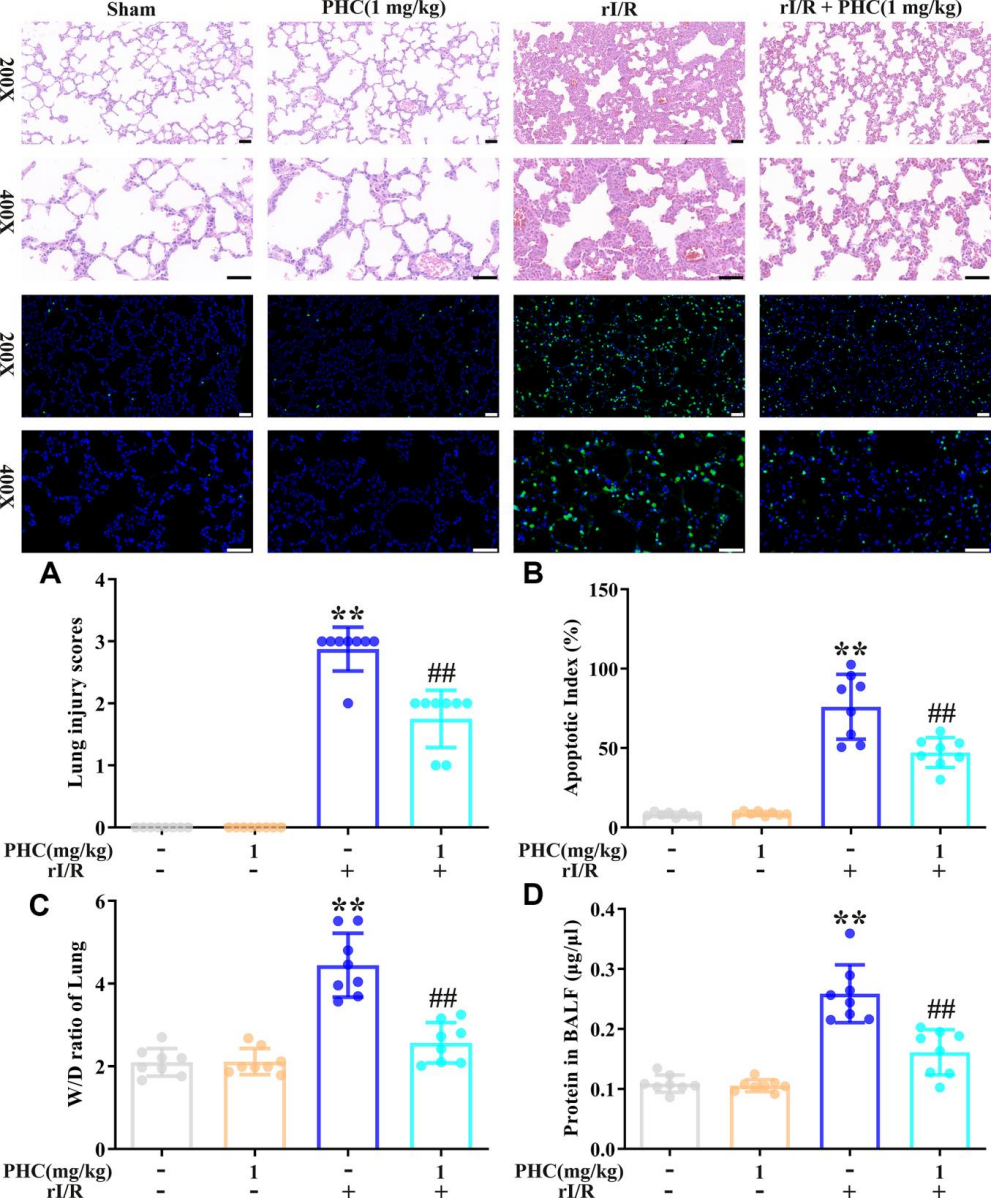
**Effects of PHC on rI/R-induced lung inflammation in rats.** (**A**) Twenty-four hours after rI/R treatment, lung sections from each investigational group (sham, PHC [1 mg/kg], rI/R, and rI/R + PHC [1 mg/kg]; n = 8) were obtained, sectioned, stained with H&E and assessed for apoptosis (original magnification 200×, 400×). A scale from 0 to 3 was used to calculate the mean lung injury score. (**B**) The relative apoptotic index (%) in the lungs was determined by TUNEL analysis. (**C**) The wet/dry weight ratios of the lungs were measured. (**D**) A BCA assay was used to determine the total protein level in BALF. Data are presented as the mean ± S.D. (n = 8, Scale bar: 50 μm). *P < 0.05, **P < 0.01 vs. the sham group. ^#^*P* < 0.05, ^##^*P* < 0.01 vs. the rI/R group.

There is substantial evidence that the strong anti-inflammatory effects of PHC are not solely due to its prevention of NF-κB and caspase-1/IL-1β signaling [34, 35]. Considering the interrelationship between anti-inflammatory signaling and Nrf2 activity, we investigated whether PHC exerted anti-inflammatory effects by inducing the Nrf2 pathway. NR8383 cells were stimulated with PHC (5 μg/mL) for 1 h and then treated with serum from rI/R rats for 24 h. PHC attenuated the production of pro-inflammatory enzymes (cyclooxygenase-2 [COX-2] and inducible nitric oxide synthase [iNOS]) in rI/R serum-treated NR8383 cells (Figure 3A). In addition, PHC stimulation prior to rI/R serum treatment increased the levels of Nrf2 pathway proteins in NR8383 cells, indicating that PHC exerted anti-inflammatory effects by upregulating the Nrf2 pathway (Figure 3B). In addition, we employed brusatol (an antagonist of Nrf2) to suppress the PHC-induced activation of Nrf2 in NR8383 cells (Figure 4C). We also explored the effects of brusatol on the levels of antioxidant enzymes induced by Nrf2 (GCLM, HO-1, NQO1 and GCLC) (Supplementary Figure 2 in Supplementary Material). Brusatol inhibits the increased levels of antioxidant enzymes induced by PHC. Nuclear NF-κB (p65), phosphorylated (p)-IκBα and NLRP3 protein levels were remarkably induced in NR8383 cells treated with rI/R serum; however, PHC post-conditioning abolished this increment (Figure 3D, 3E). As expected, PHC failed to suppress NLRP3 expression in cells preconditioned with brusatol (Figure 3D). However, surprisingly, the inhibitory effects of PHC on proteins in the NF-κB pathway were not influenced by brusatol treatment (Figure 3E). Our findings demonstrated that PHC repressed rI/R serum-induced inflammatory responses in NR8383 cells by suppressing NLRP3 in a Nrf2-dependent manner, and by inhibiting NF-κB signaling in a Nrf2-independent manner.

### PHC diminished the extent of rI/R-induced lung inflammation

Next, we examined the effects of PHC on rI/R-induced lung inflammation in rats. Rats were divided into four group: (1) sham, (2) PHC (1 mg/kg), (3) rI/R and (4) rI/R + PHC (1 mg/kg). We examined the morphology of the left lobe of the lung 24 h after rI/R stimulation. The rI/R treatment pathologically transformed the lungs, as evidenced by pulmonary hyperemia and inflammation. Post-treatment with PHC notably attenuated these alterations (Figure 4). We also calculated the lung damage score and apoptotic index (%) to evaluate the severity of the pulmonary damage (Figure 4A, 4B). As indicated in Figure 4A, 4B, the rI/R group that underwent HE staining and TUNEL staining had the most noticeable lung injury, higher lung damage scores, increased apoptosis levels, and higher apoptotic index (%) compared with the sham group (*P* < 0.05). However, the groups post-conditioned with PHC (1 mg/kg) had less injury, lower lung damage scores, decreased apoptosis levels, and lower apoptotic index (%) (*P* < 0.05). The wet/dry ratio of the lungs and the total protein level in bronchoalveolar lavage fluid (BALF) revealed analogous findings (Figure 4C, 4D). These results suggested that PHC dramatically ameliorated rI/R-induced pathological alterations and inflammation in the lungs.

### PHC attenuated rI/R-induced oxidative stress in the lungs

**Figure 5:**
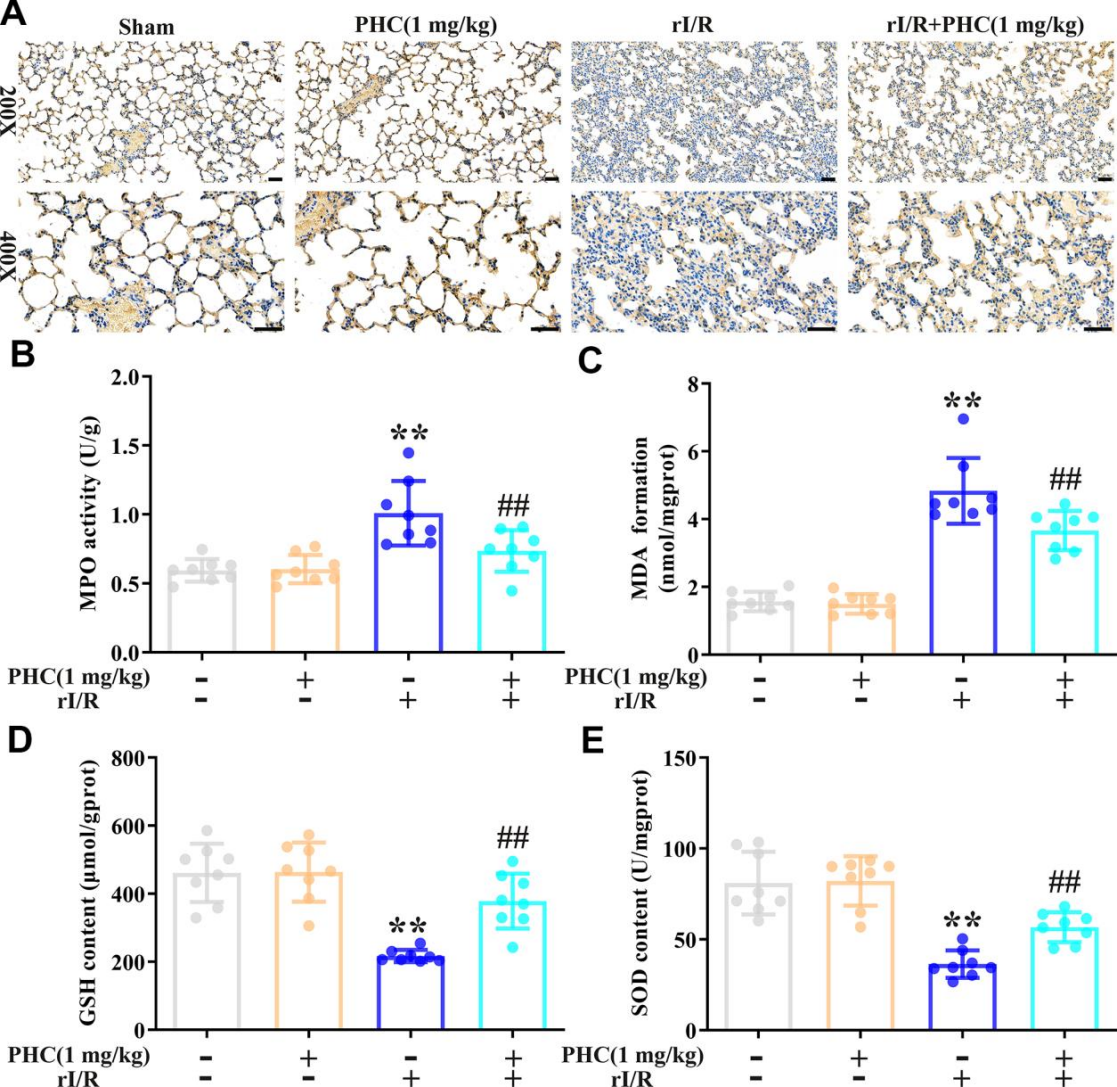
**Effects of PHC on rI/R-induced lung oxidative stress in rats.** (**A**, **B**) Myeloperoxidase (MPO) production in the lungs was analyzed using immunohistochemistry and an ELISA. (**C**–**E**) ELISAs were used to analyze malondialdehyde (MDA), glutathione (GSH) and SOD concentrations in lung homogenates. Data are presented as the mean ± S.D. (n = 8, Scale bar: 50 μm). *P < 0.05, **P < 0.01 vs. the sham group. ^#^*P* < 0.05, ^##^*P* < 0.01 vs. the rI/R group.

To determine whether PHC inhibited rI/R-induced lung inflammation by exerting antioxidative effects, we assessed the lungs for oxidative stress. We found that rI/R induced myeloperoxidase and malondialdehyde levels and reduced glutathione and superoxide dismutase (SOD) levels, while post-treatment with PHC significantly suppressed these effects (Figure 5A–5E). These findings indicated that PHC attenuated pulmonary damage by reducing oxidative stress.

### PHC ameliorated rI/R-induced inflammatory molecule and cell accumulation

**Figure 6:**
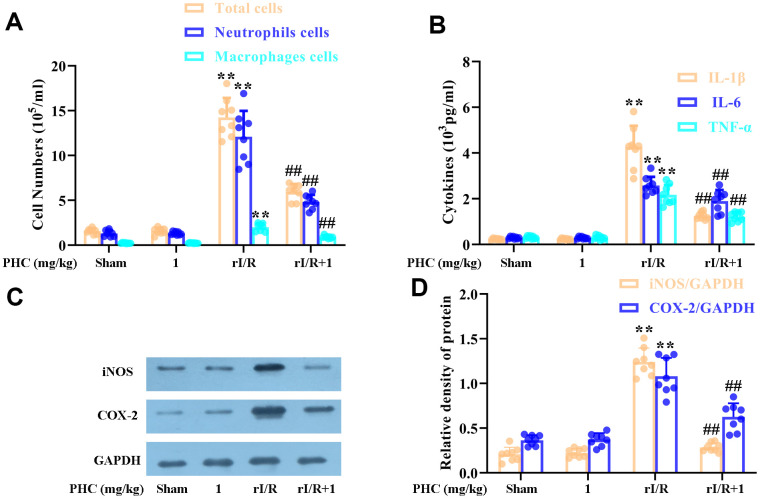
**Effects of PHC on rI/R-induced lung inflammatory cell infiltration in rats.** (**A**) A hemocytometer was used to count the numbers of total cells, neutrophils and macrophages as a measure of inflammatory cell infiltration. (**B**) ELISAs were used to measure the concentrations of pro-inflammatory molecules (IL-1β, IL-6 and TNF-α) in BALF. (**C**, **D**) Lung sections were collected 24 h after rI/R treatment, and total protein was obtained from lung homogenates. Western blotting was used to measure the levels of COX-2 and iNOS. GAPDH was used as an internal control. Data are presented as the mean ± S.D. (n = 8). *P < 0.05, **P < 0.01 vs. the sham group. ^#^*P* < 0.05, ^##^*P* < 0.01 vs. the rI/R group.

We next evaluated the number of inflammatory cells in the lungs, and found that rI/R notably augmented the inflammatory cell count in BALF, whereas PHC post-treatment remarkably reduced it (Figure 6A). Additionally, PHC attenuated IL-1β, IL-6 and tumor necrosis factor alpha (TNF-α) levels in BALF (Figure 6B), as well as iNOS and COX-2 levels in the lungs (Figure 6C, 6D). Thus, PHC ameliorated pulmonary damage by suppressing inflammatory molecule and cell accumulation following rI/R.

### PHC upregulated the Nrf2/AMPK pathways and associated antioxidant enzymes in the lungs following rI/R

**Figure 7:**
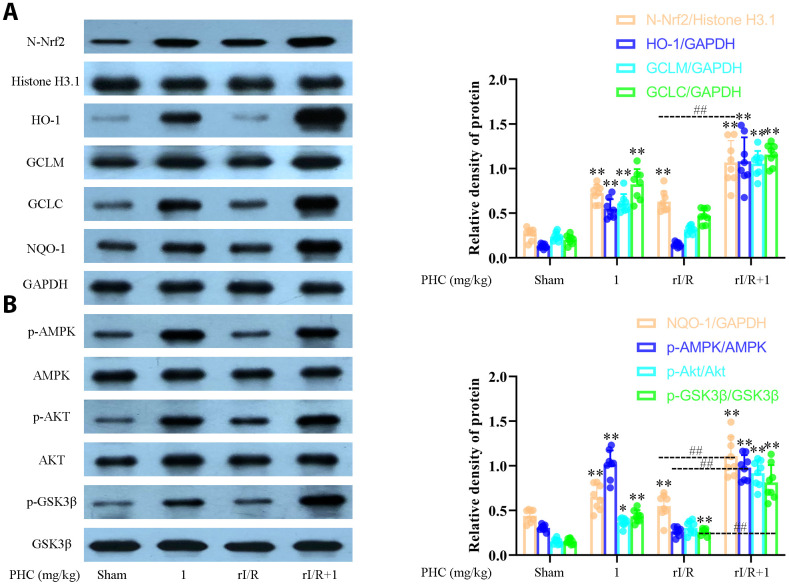
**Effects of PHC on rI/R-induced Nrf2/AMPK signaling in the lungs of rats.** Lung sections were collected 24 h after rI/R treatment. Lung homogenization was used to obtain the total protein, nuclear and cytoplasmic extracts. Western blotting was used to measure protein levels. (**A**) The nuclear concentration of Nrf2 and the protein levels of Nrf2 target enzymes (NQO1, GCLC, GCLM and HO-1). (**B**) The levels of p-AMPK, p-GSK3β, p-Akt, AMPK, GSK3β and Akt. GAPDH was used as an internal control for cytoplasmic and total proteins, while histone H3.1 was used as an internal control for nuclear proteins. Data are presented as the mean ± S.D. (n = 8). *P < 0.05, **P < 0.01 vs. the sham group. ^#^*P* < 0.05, ^##^*P* < 0.01 vs. the rI/R group.

The ability of Nrf2 to defend against lung inflammation has been demonstrated in multiple rat models. Interestingly, PHC administration remarkably enhanced the pulmonary levels of nuclear Nrf2 and its target enzymes following rI/R (Figure 7A). Additionally, PHC significantly elevated the phosphorylation of AMPK, GSK3β and Akt (Figure 7B). These results indicated that PHC may guard against rI/R-induced lung inflammation by activating the Nrf2/AMPK pathways.

### PHC ameliorated NF-κB and NLRP3 signaling in the lungs following rI/R

**Figure 8:**
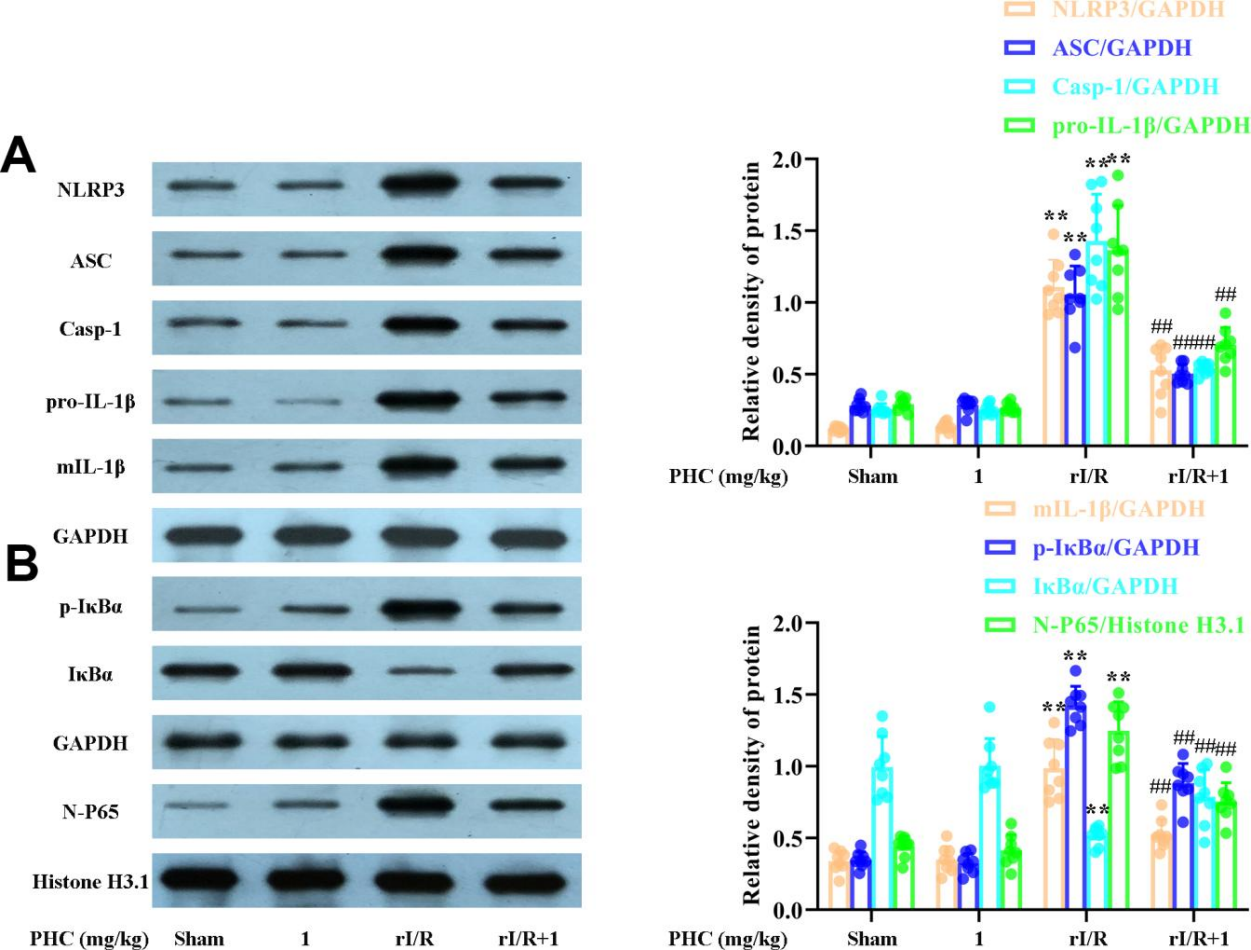
**Effects of PHC on rI/R-induced NF-κB and NLRP3 inflammasome signaling in the lungs of rats.** Lung sections were collected 24 h after rI/R treatment. Lung homogenization was used to obtain the total protein, nuclear and cytoplasmic extracts. Western blotting was used to measure protein levels. (**A**) The levels of ASC, NLRP3, pro- and mature IL-1β, and caspase-1. (**B**) The nuclear concentration of p65 and the protein levels of IκBα and p-IκBα. GAPDH was used as an internal control for cytoplasmic and total proteins, while histone H3.1 was used as an internal control for nuclear proteins. Data are presented as the mean ± S.D. (n = 8). *P < 0.05, **P < 0.01 vs. the sham group. ^#^*P* < 0.05, ^##^*P* < 0.01 vs. the rI/R group.

NF-κB and NLRP3 signaling are critical contributors to lung inflammation. Consequently, we evaluated the expression of proteins in the NF-κB and NLRP3 pathways in the lungs following rI/R. Our findings indicated that rI/R treatment remarkably elevated NLRP3, ASC and caspase-1 protein production in the lungs (Figure 8A). PHC ameliorated the rI/R-induced upregulation of NLRP3, pro-IL-1β and mature IL-1β (Figure 8A). In addition, rI/R increased cytosolic p-IκBα and nuclear NF-κB levels, while PHC suppressed these increases (Figure 8B).

### PHC inhibited oxidative stress and inflammatory responses in the lungs by activating Nrf2 following rI/R

**Figure 9:**
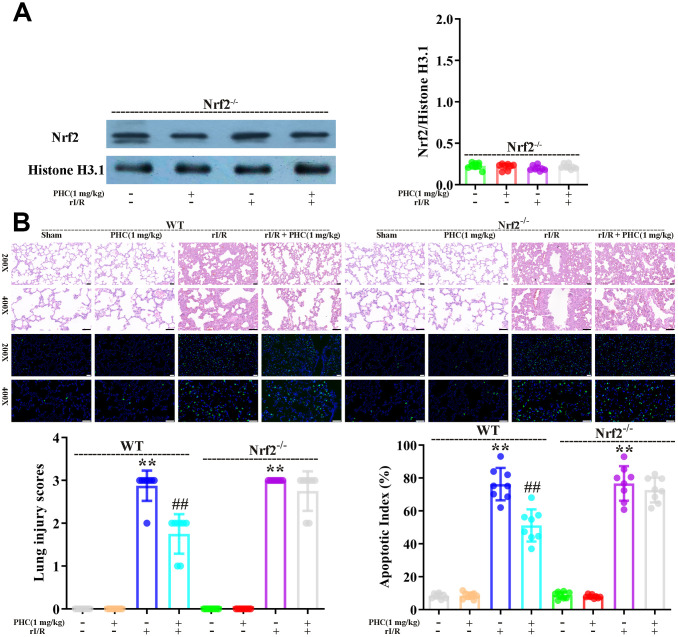
**Nrf2 dependence of the defensive effects of PHC against rI/R-induced acute lung injury in rats.** WT and Nrf2^−/−^ rats were used to model rI/R-induced acute lung injury. (**A**) To establish the depletion of Nrf2, Western blotting was used to measure nuclear Nrf2 levels in lung homogenates. Histone H3.1 was used as an internal control for nuclear proteins. (**B**) Lung sections from each investigational group (n = 8) were collected 24 h after rI/R treatment. H&E staining was used for histological assessment, and the apoptotic index (%) was calculated (original magnification 200×, 400×). A scale from 0 to 3 was used to determine the mean lung injury score. Data are presented as the mean ± S.D. (n = 8, Scale bar: 50 μm). *P < 0.05, **P < 0.01 vs. the sham group. ^#^*P* < 0.05, ^##^*P* < 0.01 vs. the rI/R group.

**Figure 10:**
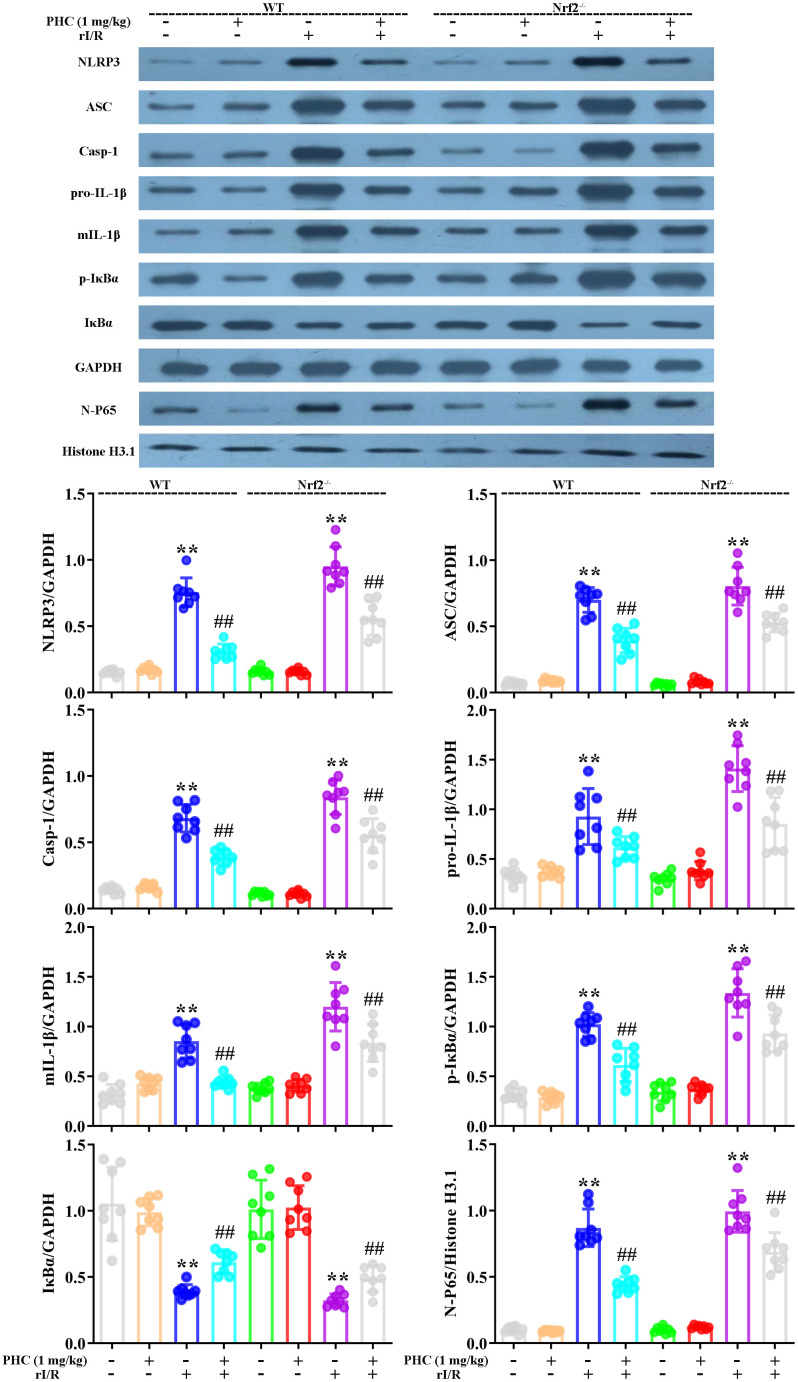
**Nrf2 dependence of the effects of PHC on NF-κB/NLRP3 signaling in the lungs following rI/R in rats.** WT and Nrf2^−/−^ rats were used to model rI/R-induced acute lung injury, and lung sections were obtained 24 h after rI/R treatment. Lung homogenization was used to obtain the total protein, nuclear and cytoplasmic extracts. Western blotting was used to measure the protein levels of ASC, NLRP3, pro- and mature IL-1β, caspase-1 p10, IκBα and p-IκBα, along with the nuclear concentration of p65. GAPDH was used as an internal control for cytoplasmic and total proteins, while histone H3.1 was used as an internal control for nuclear proteins. Data are presented as the mean ± S.D. (n = 8). *P < 0.05, **P < 0.01 vs. the sham group. ^#^*P* < 0.05, ^##^*P* < 0.01 vs. the rI/R group.

To clarify whether the activation of Nrf2 was responsible for the antioxidative and anti-inflammatory effects of PHC in the lungs following rI/R, we assessed the lungs of Nrf2^−/−^ and wild-type (WT) rats. Western blotting of nuclear fractions from the lungs confirmed the knockout of Nrf2 (Figure 9A). Histological examination revealed that PHC remarkably mitigated the rI/R-induced histopathological alterations in the lungs of WT rats, but only moderately inhibited these changes in Nrf2^−/−^ rats (Figure 9B). Likewise, PHC greatly ameliorated the rI/R serum-induced upregulation of ROS levels in primary alveolar macrophages from WT rats, but not from Nrf2^−/−^ rats (Supplementary Figure 3 in Supplementary Material), indicating that the antioxidative effects of PHC depended on the activation of Nrf2. The ability of PHC to suppress NLRP3 signaling in the lungs was significantly lower in Nrf2^−/−^ rats than in WT rats; in fact, PHC increased the production of NLRP3 pathway members in Nrf2^−/−^ rats, although the cause remains uncertain (Figure 10). On the other hand, the ability of PHC to inhibit NF-κB signaling in the lungs was maintained in Nrf2^−/−^ rats (Figure 10). These results reinforced the notion that PHC ameliorated rI/R-induced lung inflammation by inhibiting the NF-κB pathway in a Nrf2-independent manner and by suppressing the NLRP3 pathway in a Nrf2-dependent manner.

## DISCUSSION

**Figure 11:**
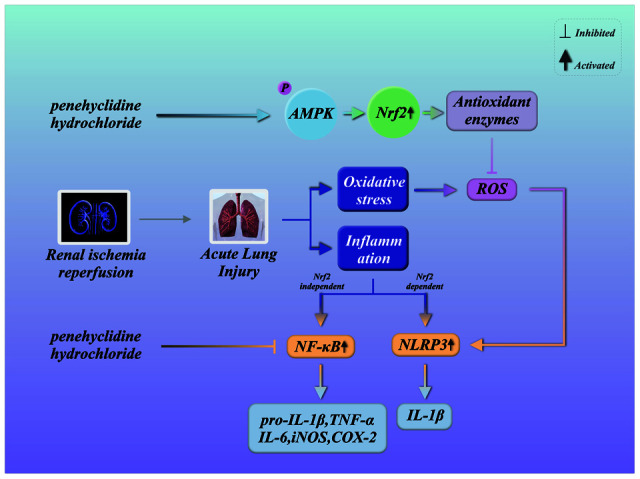
**Summary of the defensive effects of PHC against rI/R-induced acute lung injury, and the potential pathogenesis.** PHC greatly ameliorated rI/R-induced acute lung injury by suppressing oxidative stress and inflammation. These effects of PHC were linked to its stimulation of the ARE/Nrf2/AMPK pathways and its inhibition of NF-κB and NLRP3 signaling. However, the mitigation of NF-κB and NLRP3 signaling by PHC appeared to be Nrf2-independent and Nrf2-dependent, respectively.

Oxidative stress is a sign of redox imbalance and is thought to be associated with inflammatory responses [36]. Oxidative stress has been observed in various diseases characterized by systematic inflammatory responses and lung dysfunction [37, 38]. Nrf2 is an essential element of the systemic defense against oxidative stress and the associated inflammatory responses [39, 40]. PHC, an intravenous anesthetic, exhibits antioxidative and anti-inflammatory capacities that may result from its activation of Nrf2 signaling. The current investigation revealed that PHC protected against lung inflammation by inducing Nrf2 and suppressing NF-κB and NLRP3 signaling (Figure 11).

Cell models treated with serum from rI/R rats are frequently used to replicate the oxidative stress environment of lung inflammation [30, 41]. Excessive ROS production activates macrophages, neutrophils and endothelial cells and induces pulmonary damage by causing morphological alterations, protein efflux and pulmonary edema [42–44]. It is widely acknowledged that Nrf2 activation dramatically reduces ROS production and redox damage by upregulating ARE-containing genes [45]. Nrf2 also remarkably ameliorates lung inflammation and is regarded as an underlying resistance gene in diverse lung inflammation models [46, 47]. In the present study, PHC enhanced the expression of nuclear Nrf2 and its target antioxidant enzymes. Moreover, PHC prevented the massive ROS overproduction, SOD/glutathione depletion, myeloperoxidase/malondialdehyde formation and tissue damage in the lungs following rI/R. Thus, the PHC-induced activation of the Nrf2 pathway may have ameliorated pulmonary damage by abolishing excessive ROS production.

The energy sensor AMPK, which regulates cell death and survival [48], can also be stimulated by PHC and exert cellular defensive functions. A recent study in human endothelial cells revealed that Nrf2 is a downstream signal of AMPK [49]. Certain drugs that stimulate the phosphorylation of AMPK subsequently enhance the Akt-induced activation of GSK3β, ultimately activating Nrf2 [50]. Our findings indicated that PHC enhanced the phosphorylation of Akt, GSK3β and AMPK in the lungs of rats. A previous investigation suggested that CC, the only obtainable cell-permeable AMPK antagonist, may also exert AMPK-independent effects [51]. However, the data of current research recommended that PHC activated Nrf2 in an AMPK-reliant manner in macrophages through the application of CC. Meanwhile, the dependence of Nrf2 upregulation on AMPK revealed that oxidative stress may be associated with deranged energy metabolism and thus promote lung inflammation [52]; however, PHC suppressed oxidative stress and energy metabolism disruptions and thus prevented lung inflammation.

Continuous and unrestrained inflammatory responses are associated with rI/R-induced lung inflammation and oxidative stress [29–31, 41]. Upon rI/R, increased pro-inflammatory cell infiltration and molecule production elevate ROS generation, thus inducing further inflammatory responses that damage pulmonary structures [31]. The extent of lung inflammation is determined by the interdependent processes of inflammatory cell infiltration and inflammatory molecule production [42]. PHC notably suppressed rI/R-induced inflammatory responses such as leukocyte infiltration and pro-inflammatory enzyme/molecule overexpression in the lungs. Interestingly, aside from the antioxidative capacity of PHC, the ability of PHC to downregulate these inflammatory components seemed to be responsible for its mitigation of lung damage.

Among the various inflammatory pathways in the body, the NLRP3 and NF-κB pathways are prominent contributors to lung damage [53–55]. The transcription factor NF-κB, which is largely composed of the p65/p50 heterodimer, is a critical regulator of inflammatory molecules [56]. Inactivated NF-κB is located in the cytosol, combined with its antagonist IκB [56]. However, when IκB is phosphorylated and degraded, NF-κB is released, phosphorylated and moved to the nucleus, where it stimulates the transcription of its target genes [57]. The activation of NLRP3 vitally depends on the activation of NF-κB, and also can be stimulated by the production of ROS during NF-κB-induced inflammatory responses [58]. When NLRP3 is stimulated, it promotes ASC accumulation, caspase-1 sensitization and pro-IL-18/pro-IL-1β maturation [59]. Various independent pathways work simultaneously to activate molecules such as IL-1β, thus accelerating lung inflammation. The current study indicated that PHC suppressed both NLRP3 and NF-κB signaling. This double suppression may have inhibited diverse procedures in the signaling cascades responsible for rI/R-induced lung inflammation.

A previous investigation demonstrated that the activation of Nrf2 could restrain inflammatory response-induced damage by upregulating antioxidant pathways [60]. Nevertheless, the association of Nrf2 with inflammatory signals such as NLRP3 and NF-κB has also been disputed [61]. We further studied the relationships among these signals. Preconditioning with brusatol lessened the ability of PHC to suppress ROS production and enhance cell viability in NR8383 cells. The PHC-induced blockage of ROS generation was also hindered in primary alveolar macrophages from Nrf2^−/−^ rats. In brusatol-stimulated NR8383 cells and Nrf2^−/−^ rat alveolar macrophages, the inhibition of NLRP3 by PHC was distinctly lessened, whereas the inactivation of IκBα by PHC was not influenced. Thus, although PHC seemed to mitigate rI/R-induced lung inflammation by activating Nrf2 and suppressing NF-κB and NLRP3 signaling, PHC may have repressed these inflammatory signals by independent mechanisms. NF-κB inactivation has not been proven to be sufficient to attenuate rI/R-induced lung inflammation. On the other hand, PHC administration clearly ameliorated the morphological alterations in the lungs of WT rats following rI/R, while this defensive action was not fully reversed in Nrf2^−/−^ rats. These findings revealed that the anti-inflammatory effects of PHC were not completely dependent on Nrf2. It is also possible that other priming pathways besides NF-κB induce NLRP3 signaling and contribute to rI/R-induced acute lung injury.

In summary, PHC notably ameliorated rI/R-induced lung inflammation by inhibiting inflammatory damage and oxidative injury in rats. PHC exerted these effects by stimulating the Nrf2/AMPK pathways and restricting NLRP3 and NF-κB signaling. The antioxidative actions of PHC mainly resulted from its activation of Nrf2, while the anti-inflammatory effects of PHC during rI/R appeared to be both Nrf2-dependent and -independent. These findings support the use of PHC to treat rI/R-induced lung inflammation.

## MATERIALS AND METHODS

### Medications and chemicals

PHC was purchased from Lisite Corporation (Chengdu, China). The t-BHP, brusatol (Nrf2 inhibitor) and CC (AMPK inhibitor) were purchased from Invitrogen-Gibco (Grand Island, NY, USA). F-12K medium, fetal bovine serum, dimethyl sulfoxide, penicillin and streptomycin were purchased from Sigma-Aldrich (St. Louis, MO, USA). Malondialdehyde, myeloperoxidase, SOD and glutathione assay kits were supplied by Biolegend (San Diego, CA, USA). Rat IL-6, IL-1β and TNF-α enzyme-linked immunosorbent assay (ELISA) kits were obtained from the Jiancheng Bioengineering Institute of Nanjing (Jiangsu, China). Horseradish peroxidase-conjugated anti-rabbit IgG was purchased from Cell Signaling (Boston, MA, USA). Antibodies against IL-1β, caspase-1, ASC, NLRP3, IκBα, p-IκBα, GSK3β, p-GSK3β, AMPK, p-AMPK, GCLC, GCLM, NQO1, HO-1, Nrf2, GAPDH and histone H3.1 were purchased from Protein-Tech (Boston, MA, USA). Unless otherwise specified, all other reagents were purchased from the Jiancheng Bioengineering Institute of Nanjing.

### Cell culture

NR8383 cells, a rat alveolar macrophage-derived cell line, were purchased from the Cell Bank of the Chinese Academy of Sciences (Shanghai, China). F-12K medium containing 50 U/mL penicillin, 50 μg/mL streptomycin and 20% fetal bovine serum was used to culture the cells in a humidified atmosphere with 5% CO_2_ and 95% air.

### Viability assay

A CCK8 assay was used to measure NR8383 cell viability. NR8383 cells (1 × 10^4^ cells per well) were cultured in a 96-well plate at 37°C for 24 h. The cells were stimulated with PHC (1, 2.5 or 5 μg/mL) with or without brusatol for 24 h, and were subsequently treated with t-BHP (10 mM) for 4 h. CCK8 (20 μL) was applied to the cells for 2 h, and a microplate reader was used to determine the absorbance at 450 nm. NR8383 cell viability was expressed as a percentage of that in the control group.

### ARE promoter activity

A 96-well plate was used to culture NR8383 cells (1.5 × 10^4^ cells per well). When the cells reached 75% confluence, they were transfected with plasmids (pGL4.37 and pGL4.74). PHC was administered at various levels (1, 2.5 or 5 μg/mL) for 24 h, or at 5 μg/mL for 4, 8, 16 and 24 h. Then, the ARE-induced promoter activity was measured on a dual-luciferase reporter examination system.

### Animal care

Animal experiments were performed on WT and Nrf2^−/−^ Sprague Dawley rats (180-250 g, 10 weeks old). The investigations were certified by the Animal Use Committee of Shandong University. The rats were provided free access to irradiated food and sterile acidified water, and were housed in standard approved experimental cages prior to the investigations.

### Generation of the rat model of rI/R-induced lung inflammation

To create a rat model of rI/R-induced lung inflammation, we induced renal ischemia by bilaterally occluding the renal pedicles for 1 h. The return of blood flow was used to confirm renal reperfusion when the clamp was removed. The same operation without bilateral occlusion was performed in the sham rats. A warmed cage was used to recover the rats. Certain rats were intravenously administered PHC 30 min after reperfusion. WT and Nrf2^−/−^ rats were each randomly assigned into four groups: (1) sham, (2) PHC (1 mg/kg), (3) rI/R and (4) rI/R + PHC (1 mg/kg). Diethyl ether was used to sacrifice the rats 24 h after rI/R.

### Isolation of alveolar macrophages

Phosphate-buffered saline (PBS, 6 mL) was used to perform lung lavage through a tracheal tube 10 times. A 50-mL centrifuge tube used to transfer and collect the lavage fluid. The tube was centrifuged for 8 min at 130 x *g*, and then the supernatant was discarded. Red blood cell lysis buffer (3 mL) was added, and then the lavage fluid was centrifuged for 10 min at 440 x *g*. PBS was used to suspend the precipitate and adjust the cell concentration to 2-3×10^6^ cells/mL. Giemsa staining was used to detect the purity of the cells. A trypan blue exclusion assay was used to detect cell activity, and a hemocytometer was used to count the cells.

### Assessment of morphological injury

For the morphological assessment of lung samples, the rats were euthanized and their left lung lobes were fixed with formalin (4%) and dehydrated with ethanol. The specimens were then embedded and sliced in paraffin blocks, and hematoxylin and eosin (H&E) staining was applied to 5-μm sections as described previously. A light microscope was used to observe pathological alterations in the H&E-stained sections. An approved evaluation procedure was used to analyze lung inflammation. Specifically, pulmonary damage was assessed based on hemorrhaging, neutrophil infiltration, edema, bronchiolar epithelial exfoliation and alveolar hyalinization, and five visual fields were examined for each slice. The investigations were carried out blindly. The severity of pulmonary damage was scored on a scale from 0 to 3: (0) no injury, (1) mild injury, (2) moderate injury and (3) severe injury.

### Wet/dry weight ratios

The wet weights of the lung samples were determined, and then the lungs were dried in an oven for three days at 60°C so that the dry weights could be measured. The extent of pulmonary edema was determined based on the wet/dry weight ratio.

### Immunohistochemistry analysis

Commercial immunohistochemistry kits (Beyotime, Haimen, China) were used to visualize the MPO activity in the lung sections as described by the manufacturer’s directions. Briefly, the sample sections were heated for 1.5 h at 65-75 °C. Immersion in xylene was used to dewax the segments for 10 min. A graded ethanol series was used to rehydrate the segments each for 5 min. Triton X-100 (0.5%) was used to permeabilize the sections at 25 °C for 20 min following antigen recovery. The sections were incubated in a freshly prepared 3% hydrogen peroxide blocking solution at 25 °C for 10 min to eliminate the intrinsic peroxidase activity. The sections were incubated in the primary anti-MPO antibodies (1: 50) at 4 °C overnight after blocking, and then, the sections were incubated in the appropriate secondary antibodies (1: 100) at 25 °C for 1 h. A microscope was used to visualize the staining. Hematoxylin was used to restain the washed sections for 3 min, and then, hydrochloric acid/alcohol was used to dehydrate the segments. The microscope was used to visualize the rinsed, dehydrated segments.

### Determination of lung apoptosis

A TUNEL evaluation was used to assess lung apoptosis with a cell death assay kit as described previously. The TUNEL-positive nuclei present green fluorescence, and the nuclei of whole lung samples present blue fluorescence; the degree of apoptosis is reported as the quantity of apoptotic cells/the whole number of cells measured ×100%.

### Evaluation of BALF

The rats were sacrificed, and then the trachea was exposed. BALF was obtained from the tracheal liquid, which was acquired through the injection and aspiration of PBS (0.5 mL) three times. Pelleted cells were obtained via centrifugation of the BALF, and the total protein level in the supernatant was determined by the bicinchoninic acid (BCA) method. ACK Lysis Buffer was used to lyse the cells for 5 min, and then the cells were washed twice with ice-cold PBS. Then, the cells were centrifuged at 4°C for 10 min at 3,000 rpm, and the sedimented cells were resuspended in PBS. A hemocytometer was used to count the total cell number, and Wright-Giemsa staining was used to count the differential inflammatory cell number. ELISAs were used to determine the levels of inflammatory proteins in the supernatants, which were stored at −80°C.

### Wright-Giemsa staining

Cells from BALF were spun onto glass slides. Methyl alcohol was applied to the slides, and Wright-Giemsa solutions A and B were added to the slides for 1 min and 5 min, respectively. Running water was used to moderately wash off the dye, and a microscope was used to observe the drying samples. The number of differential inflammatory cells in a total of 300 cells was counted in each microscopic field, and the average cell number was calculated.

### ELISA

The levels of IL-1β, IL-6 and TNF-α in BALF were determined using ELISA kits. A microplate reader was used to read the absorbance at 450 nm.

### Myeloperoxidase, malondialdehyde, glutathione and SOD activity analysis

The excised lungs were homogenized in saline. ELISA kits were used to measure myeloperoxidase, malondialdehyde, glutathione and SOD production in accordance with the kit instructions.

### Determination of ROS production

2’, 7’-Dichlorofluorescein diacetates (DCFH-DA), an oxidant-susceptible fluorescent probe, was used to determine the ROS-eliminating capacity of PHC. NR8383 cells were treated with PHC for 24 h with or without brusatol before being treated with t-BHP (10 mM) for 5 min. Alternatively, primary alveolar macrophages were pre-stimulated with PHC (5 μg/mL) for 1 h and then treated with serum from rI/R rats for 24 h. Then, DCFH-DA (5 μM) was used to stain the PHC-stimulated cells for 40 min. A microplate reader was used to detect the fluorescence activity at wavelengths of 488 nm for excitation and 535 nm for emission.

### Separation of cytoplasmic and nuclear extracts

An NE-PER Nuclear and Cytoplasmic Extraction Reagents Kit was used to prepare nuclear and cytosolic fractions according to the manufacturer’s manual. Ice was applied during all procedures.

### Western blot analysis

For protein extraction, radioimmunoprecipitation assay buffer containing protease and phosphatase inhibitors was used to lyse the homogenized lung samples and stimulated cells for 30 min. The homogenized lung samples were then centrifuged at 12,000 rpm at 4°C for 10 min. The protein levels in the supernatants were analyzed by the BCA method. The protein samples were electrophoretically separated on a sodium dodecyl sulfate polyacrylamide gel (10-12.5%) and then transferred to a polyvinylidene difluoride membrane. Skim milk (5%) was used to block the membrane for 1 h at room temperature. The membrane was treated with each primary antibody (1:1,000) at 4°C overnight, and then treated with the corresponding secondary antibody (1:5,000) for 1 h at room temperature. An enhanced chemiluminescence Western blot detection system was used to visualize the blots. ImageJ gel analysis software was used to assess the band intensities.

### Statistical evaluation

Data were analyzed with GraphPad Prism 8.0 and presented as the mean ± standard deviation (S.D.). Differences among groups were assessed with one-way analysis of variance followed by the Least Significant Difference method. *P* < 0.05 was considered statistically significant.

## Supplementary Material

Supplementary Figures
