# Supplementary material for: Penehyclidine hydrochloride inhibits renal ischemia/reperfusion-induced acute lung injury by activating the Nrf2 pathway
Source: Aging (Albany NY). 2020 Jul 11;12(13):13400–21. doi: 10.18632/aging.103444 (PMC7377887; doi:10.18632/aging.103444)
Supplement: Supplementary Figures [file aging-12-103444-s001..pdf]

SUPPLEMENTARY FIGURES

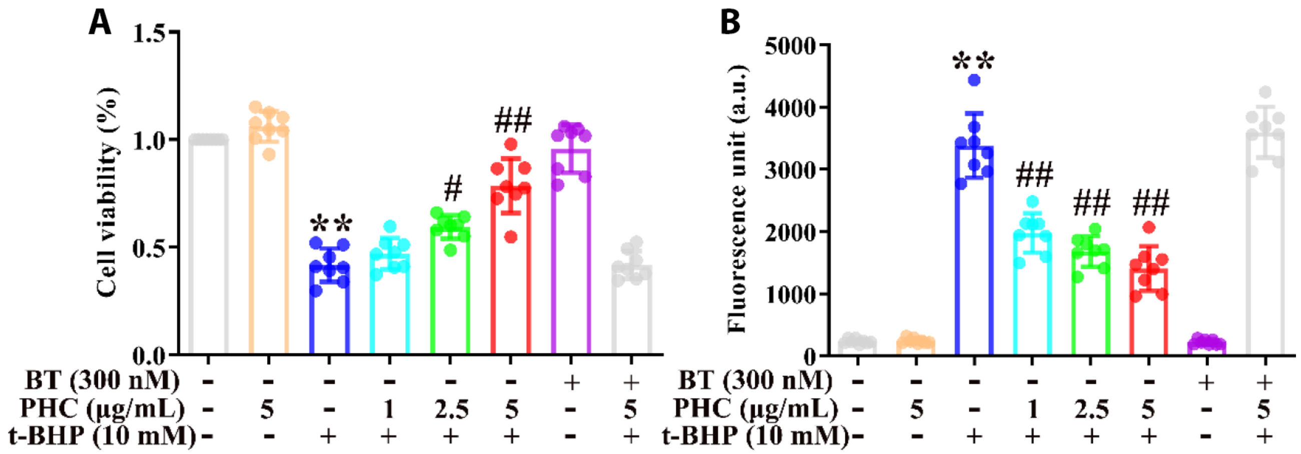

Supplementary Figure 1. Effects of PHC and brusatol on t-BHP-induced oxidative damage in NR8383 cells. (**A**) NR8383 cells were stimulated with PHC (1, 2.5, 5 μg/mL) for 24 h with/without brusatol (300 nM), and then were treated with t-BHP (10 mM) for 4 h. A CCK8 assay was used to measure cell viability. (**B**) NR8383 cells were treated with PHC (1, 2.5, 5 μg/mL) for 24 h with/without brusatol, stained with DCFH-DA (5 μM) for 40 min and then treated with t-BHP (10 mM) for 5 min to produce ROS. A fluorescence microplate reader was used to measure ROS levels. Data are presented as the mean ± S.D. (n = 8). *P < 0.05, **P < 0.01 vs. the control group. $^{\#}P$ < 0.05, $^{\#\#}P$ < 0.01 vs. the t-BHP group.

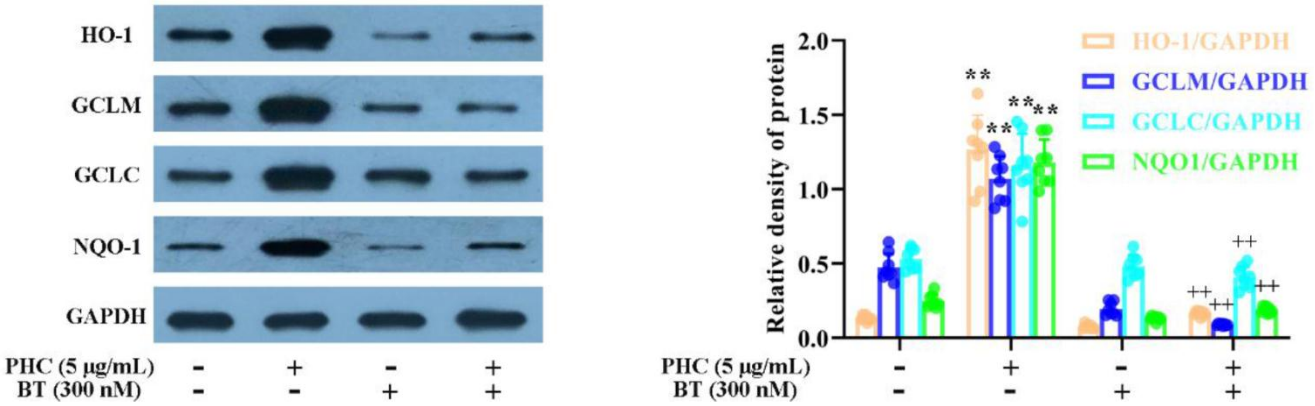

Supplementary Figure 2. Suppression of Nrf2-induced antioxidant enzymes by brusatol. NR8383 cells were stimulated with brusatol (an antagonist of Nrf2, 300 nM) for 1 h and then exposed to PHC (5 μg/mL) for 1 h before being treated with serum from rI/R rats for 24 h. Western blotting was used to measure the protein levels of GCLM, HO-1, NQO1 and GCLC. GAPDH was used as an internal control. Data are presented as the mean ± S.D. (n = 8). *P < 0.05, **P < 0.01 vs. the control group. ^{+}P < 0.05, ^{++}P < 0.01 vs. the PHC alone group.

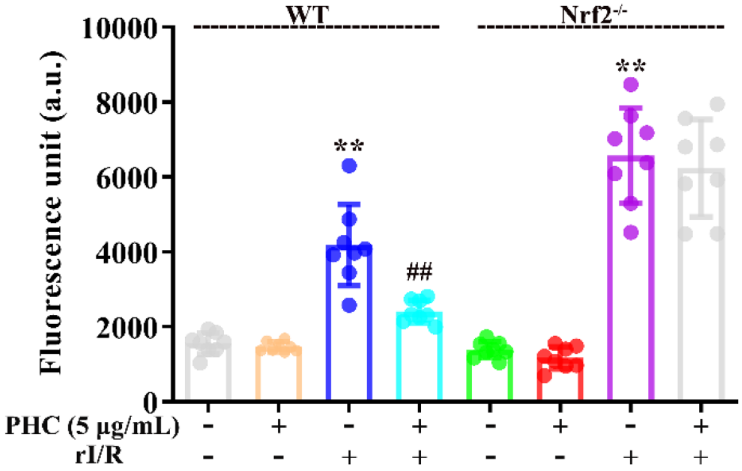

Supplementary Figure 3. Nrf2 dependence of the antioxidative effects of PHC in primary rat alveolar macrophages. Alveolar macrophages isolated from WT and $Nrf2^{-/-}$ rats were pre-stimulated with PHC (5 μg/mL) for 1 h and then treated with serum from rI/R rats for 24 h. Subsequently, the cells were stained with DCFH-DA (5 μM) for 40 min, and a fluorescence microplate reader was used to determine ROS levels. Data are presented as the mean ± S.D. (n = 8). **P* < 0.05, ***P* < 0.01 vs. the control group. $^{\#}P < 0.05$, $^{\#\#}P < 0.01$ vs. the rI/R group.
